# eNOS-Dependent Antisenscence Effect of a Calcium Channel Blocker in Human Endothelial Cells

**DOI:** 10.1371/journal.pone.0088391

**Published:** 2014-02-10

**Authors:** Toshio Hayashi, Tomoe Yamaguchi, Yasufumi Sakakibara, Kumiko Taguchi, Morihiko Maeda, Masafumi Kuzuya, Yuichi Hattori

**Affiliations:** 1 Department of Geriatrics, Nagoya University Graduate School of Medicine, Nagoya, Japan; 2 Department of Molecular and Medical Pharmacology, Graduate School of Medicine and Pharmaceutical Sciences, Toyama, Japan; Osaka University Graduate School of Medicine, Japan

## Abstract

Senescence of vascular endothelial cells is an important contributor to the pathogenesis of age-associated vascular disorders such as atherosclerosis. We investigated the effects of antihypertensive agents on high glucose-induced cellular senescence in human umbilical venous endothelial cells (HUVECs). Exposure of HUVECs to high glucose (22 mM) for 3 days increased senescence-associated- β-galactosidase (SA-β-gal) activity, a senescence marker, and decreased telomerase activity, a replicative senescence marker. The calcium channel blocker nifedipine, but not the β_1_-adrenergic blocking agent atenolol or the angiotensin-converting enzyme inhibitor perindopril, reduced SA-β-gal positive cells and prevented a decrease in telomerase activity in a high-glucose environment. This beneficial effect of nifedipine was associated with reduced reactive oxygen species (ROS) and increased endothelial nitric oxide synthase (eNOS) activity. Thus, nifedipine prevented high glucose-induced ROS generation and increased basal eNOS phosphorylation level at Ser-1177. Treatment with *N* ^G^-nitro-L-arginine (L-NAME) and transfection of small interfering RNA (siRNA) targeting eNOS eliminated the anti-senscence effect of nifedipine. These results demonstrate that nifedipine can prevent endothelial cell senescence in an eNOS-dependent manner. The anti-senescence action of nifedipine may represent a novel mechanism by which it protects against atherosclerosis.

## Introduction

Aging is increasingly regarded as a major risk factor for the development of cardiovascular disorders, including atherosclerosis, hypertension, and their complications such as stroke and myocardial infarction [1], [2]. With increasing age, the vasculature undergoes functional and structural impairment. It is thus of up most importance to unravel the molecular and cellular mechanisms that may be potentially involved in vascular aging. Vascular aging is characterized by the transition of endothelial cells from an anti-atherosclerotic state to a proatherosclerotic one [3]. Vascular endothelial cells, among many other cell types, can undergo replicative senescence *in vitro*. Senescence is characterized by specific changes in cell morphology and gene expression, which directly correlate with an impairment of endothelial integrity and function [4]. Some of these changes in endothelial cells, including decreased endothelial nitric oxide synthase (eNOS) activity and decreased nitric oxide (NO) production, can lead to a dysregulated vascular tone and a proatherosclerotic and prothrombotic environment [3], [5], [6]. Thus, senescence of vascular endothelial cells are proposed to be proatherogenic [7], [8]. Senescent endothelial cells have been identified at sites of atherosclerotic lesions *in vivo* [9], [10].

Diabetes mellitus is a documented high risk factor for the development of atherosclerosis. Insulin resistance and hyperinsulinemia, which are essential features of type 2 diabetes, can be regarded as a premature aging syndrome in which the dysregulation of insulin/Akt signaling promotes endothelial cell senescence and leads to diabetic vascular complications [11]. We and others have documented that high glucose can induce endothelial cell senescence [12]–[14]. As patients with diabetes have a much higher rate of hypertension than would be expected in the general population, it was of interest to find out antihypertensive agent(s) which are effective against high glucose-induced endothelial cell senescence. We found that the calcium channel blocker nifedipine significantly inhibited cellular senescence in human umbilical venous endothelial cells (HUVECs). Further studies were then undertaken to gain insight into possible mechanism(s) involved in its beneficial effect on endothelial cell senescence. We also examined whether angiotensin II and oxidized low-density lipoprotein (oxLDL), which are other critical factors for atherosclerosis, can induce endothelial cell senescence.

## Materials and Methods

### Cell culture

HUVECs were purchased from Lonza Walkersville and cultured in endothelial cell growth medium-2 until the start of the experiment. The cells were cultured in modified endothelial cell growth medium-2 that lacked insulin-like growth factor-1 but contained 2% fetal bovine serum during the experimental term. According to our previous study [12], five- to seven-passage subconfluent cells were used in the experiments. Cells were harvested at subconfluence and seeded into six-well plates. They were then stimulated with different concentrations of D-glucose (5.5, 22, and 33 mM), angiotensin II (1 and 10 µM), or oxLDL (15 and 50 µg/ml) for 3 days. Atenolol, perindopril, nifedipine, *N* ^G^-nitro-L-arginine (L-NAME), N-acetylcysteine (NAC), or small interfering RNA (siRNA) targeted to eNOS was treated during the same term as high glucose. Hydrogen peroxide (H_2_O_2_) and sodium nitroprusside were added 30 min before the measurement of fluorescence.

### Senescence-Associated-β-Galactosidase (SA-β-Gal)

Cells were fixed for 10 min in 2% formaldehyde, 0.2% glutaraldehyde in PBS, and incubated for 12 hours at 37°C without CO_2_ with fresh β-gal staining solution: 1 mg/ml 5-bromo-4-chloro-3-indolyl-β-D-galactopyranoside, 5 mM potassium ferrocyanide, 5 mM potassium ferricyanide, and 2 mM MgCl_2_, pH 6.0. The cells were counterstained with 4′6-diamidinophenylindole (DAPI; 0.2 mg/ml in 10 mM NaCl) for 10 min to count the total cell number. The percentage of SA-β-gal-positive cells was determined by counting the number of blue cells within a sample of 1,000 cells. SA-β-gal activity was also measured by flow cytometry as described previously [15]. After the experiment, cells were incubated with C_12_FDG (fluorogenic substrate 5-dodecanoyl- aminofluorscein di-β-D-galactopyranoside; 33 mM) at 37°C for 30 min. Cells were trypsinized and analyzed using a FACSCalibur flow cytometer (Becton Dickinson).

### Human telomerase activity assay

The quantitative determination of telomerase activity was performed according to the manufacturer's protocol for the TeloTAGGG Telomerase PCR ELISA^PLUS^ kit (Roche Diagnostics) based on the telomere repeat application protocol (trap) assay. To measure telomerase activity, 2 mg of protein was used in the PCR. Protein concentrations were determined using a DC protein assay kit (Bio-Rad).

### Western blot analysis

Total cell lysates (10 µg protein/lane) were separated on a 12% SDS-PAGE. Separated proteins were electrophoretically transferred onto polyvinylidene fluoride membranes and were blocked for 1 hour at room temperature in Tris-buffered saline containing 5% powdered skim milk. The membranes were incubated overnight at 4°C with the corresponding primary antibody. The membranes were then washed three times with Tween-Tris-buffered saline, followed by incubation with an alkaline phosphatase-conjugated secondary antibodies at room temperature for 1 hour. Protein bounds were detected using the enhanced chemiluminescence detection system.

### Flow cytometric analysis of reactive oxygen species (ROS) and superoxide generation

Intracellular oxidant generation was detected with the fluorescent probe C6827, 5-(and-6)-chloromethyl-2′,7′-dichlorodihydrofluorescein diacetate, acetyl ester (CM-H_2_DCFDA; Invitrogen) [16]. Cells were incubated with CM-H_2_DCFDA (10 mM) at 37°C for 30 min, and flow cytometry was performed. Superoxide anion was measured using dihydroethidium (DHE; Invitrogen)

### Transfection of eNOS siRNAs

siRNAs targeting human eNOS were developed in our laboratory [17]. Nonsilencing control siRNA was used as a negative control. The following sequences were used; 5′-CGAGGAGACUUCCGAAUCUUU-3′ (sense) and 5′- PAGAUUCGGAAGUCUCCUCGUU-3′ (antisense) for eNOS siRNA; 5′-UUCUUCGAACGUGUCACGUdTdT-3′ (sense) and 5′-ACGUGACACGUUCGGAGAAdTdT-3′ (antisense) for control siRNA. siRNA (1 nM) was transfected using Lipofectamine RNAiMAX (Invitrogen). After incubation for 72 hours, the down-regulation of eNOS expression was confirmed by Western blotting.

### Statistical analysis

Data are shown as mean ± SEM. Analysis was performed with Prism (ver. 4; GraphPad Software) using 1-way ANOVA followed by Tukey's multiple comparison test when appropriate. Pairwise comparisons were performed using Student *t* test. Level of significance was established a priori at *P*<0.05.

## Results

### Effects of high glucose, angiotensin II, and oxLDL on endothelial cell senescence

**Figure 1:**
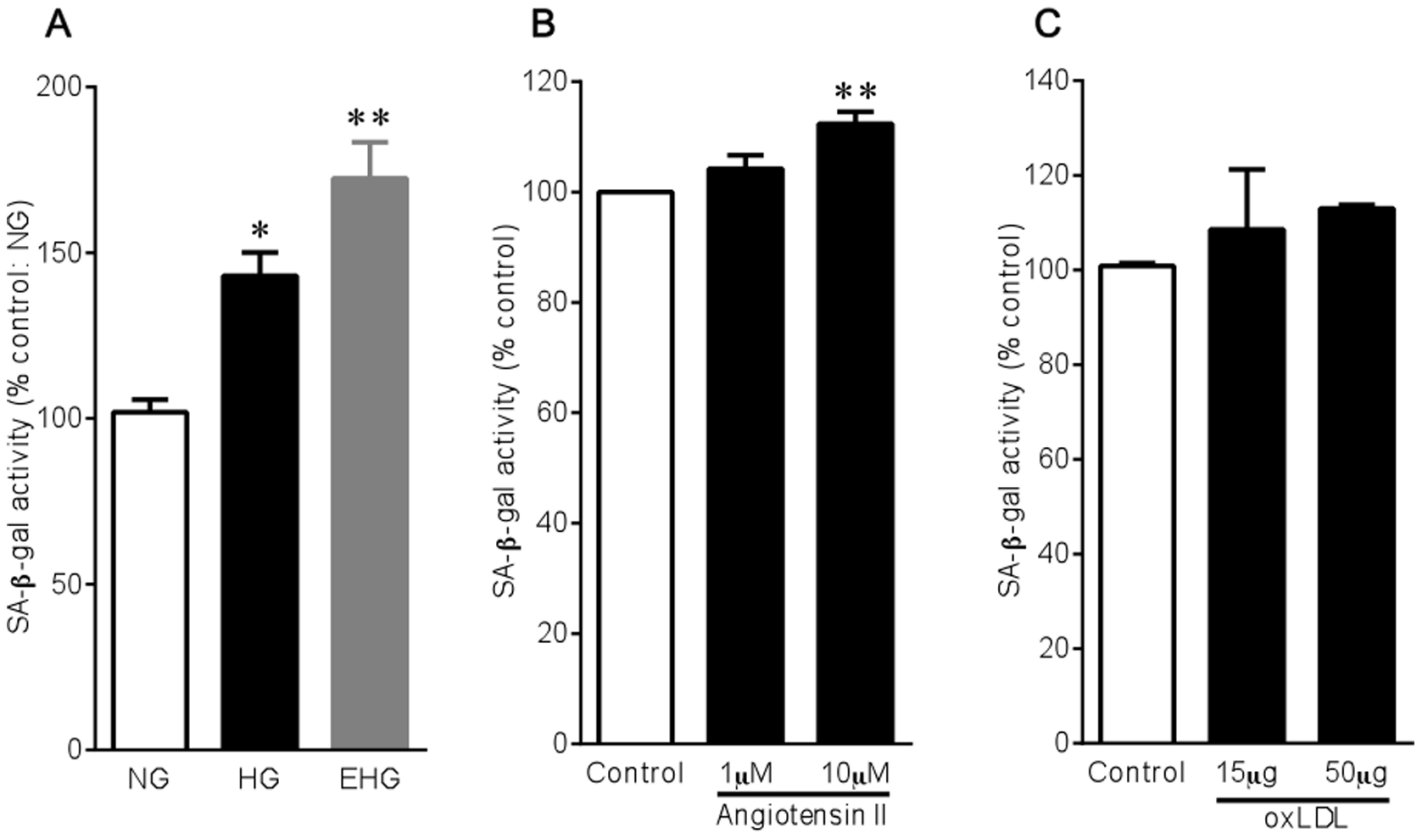
Effects of high glucose (A), angiotensin II (B), and oxLDL (C) on SA-β-gal activity in HUVECs. Cells were incubated with different glucose concentrations (NG, 5.5 mM; HG, 22 mM; EHG, 33 mM), angiotensin II (1 and 10 µM), or oxLDL (15 and 50 µg/ml) for 3 days. Bar graphs are means±SEM from 3-4 independent experiments. *P<0.05, **P<0.01 versus control.

Diabetes, hypertension, and dyslipidemia are the most common lifestyle-related diseases. High glucose, angiotensin II, and oxLDL are important in causing the development of diabetes, hypertension, and dyslipidemia, respectively. We initially examined whether they can lead to endothelial cell senescence by means of the measurement of SA-β-gal activity, a widely used quantitative marker for aging *in vitro*. Glucose increased SA-β-gal activity in HUVECs in a concentration-dependent manner (Figure 1A). When mannitol was used to rule out an osmotic effect, mannitol was found to be without effect on cellular senescence. Angiotensin II and oxLDL were also effective in increasing SA-β-gal activity, though were less marked than high glucose (Figure 1B and 1C).

### Effects of antihypertensive agents on endothelial cell senescence

**Figure 2:**
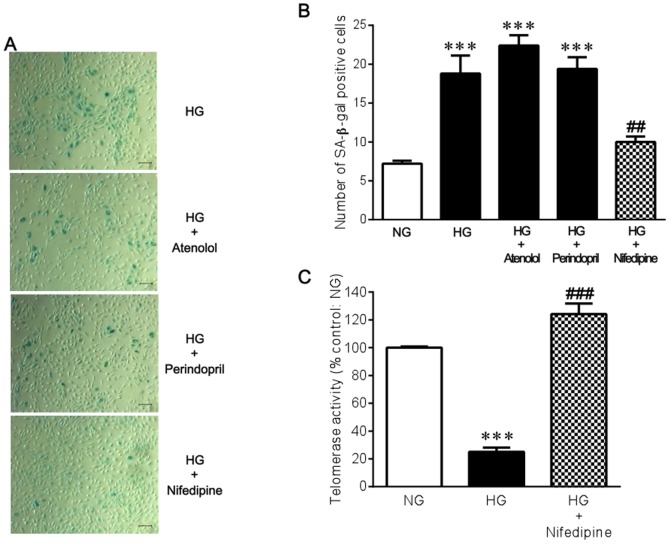
Effects of atenolol, perindopril, and nifedipine on high glucose-induced cellular senescence in HUVECs. **(A)** Cytochemical staining for SA-β-gal activity. Cells were incubated with 22 mM glucose for 3 days in the absence and presence of 10 µM atenolol, 10 µM perindopril, or 1 nM nifedipine. Bar = 100 µm. **(B)** A bar graph summarizes the results from 5 experiments shown in **A**. **(C)** Telomerase activity was measured by the telomere repeat application protocol (trap) assay (n = 4). ***P<0.001 versus NG (5.5 mM glucose). ##*P*<0.01, ###*P*<0.001 versus HG (22 mM glucose) alone.

We next investigated whether several different types of antihypertensive agents can inhibit high glucose-induced endothelial cell senescence. Neither the β_1_-adrenergic blocking agent atenolol nor the angiotensin-converting enzyme inhibitor perindopril prevented the increase in the number of SA-β-gal positive cells that was induced by high glucose conditions. In contrast, the calcium channel blocker nifedipine significantly reduced SA-β-gal positive cells under high glucose (Figure 2A and 2B). Telomerase activity was significantly decreased in a high glucose environment, and nifedipine significantly prevented the decrease in telomerase activity (Figure 2C), suggesting the possible involvement of replicative senescence in the anti-senescence action of nifedipine. However, nifedipine did not affect either SA-β-gal or telomerase activity under normal glucose conditions (Figure S1). There appeared to be an optimal concentration of nifedipine to produce the anti-senescence effect on high glucose-exposed endothelial cells. The concentrations above and below 1 nM were found to have a limited effect (data not shown). Furthermore, increasing medium concentration of Ca^2+^ from 1.8 to 3.6 mM resulted in a rather significant reduction in SA-β-gal activity under both normal and high glucose conditions (Figure S2), suggesting that the beneficial effect of nifedipine on endothelial cell senescence cannot be solely attributed to its calcium channel blocking action.

### Effect of nifedipine on high glucose-induced ROS generation

**Figure 3:**
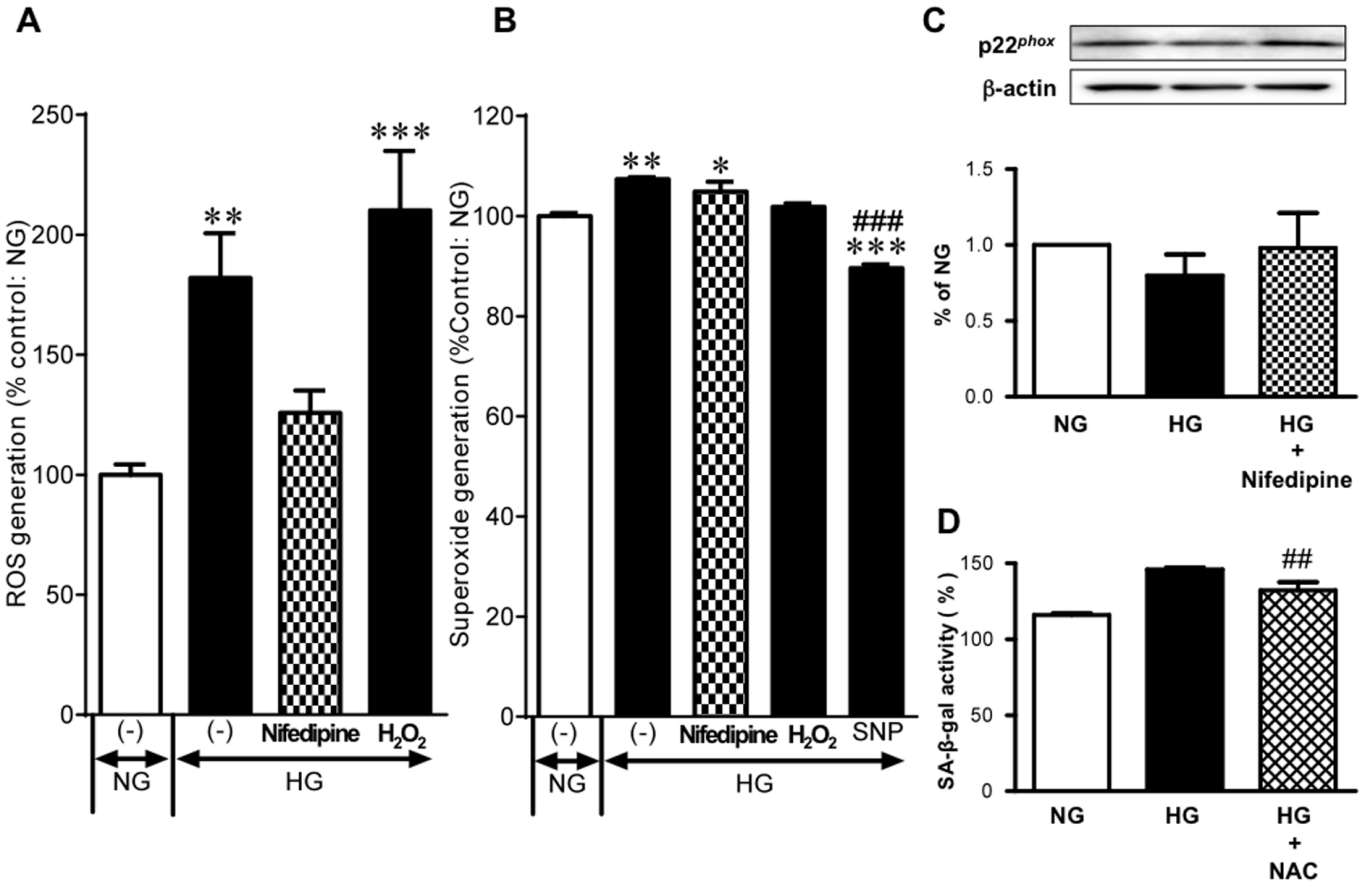
ROS generation in HUVECs exposed to high glucose. **(A)** Effects of 1 µM H_2_O_2_ on ROS generation in cells exposed to 22 mM glucose (HG) for 3 days (n = 4–6). The cells were stained with fluorescent probe CM-H_2_DCFDA, and ROS were detected by flow cytometry. **(B)** Effects of 1 nM nifedipine, 220 µM H_2_O_2_, and 500 µM sodium nitroprusside (SNP) on superoxide production in cells exposed to HG for 3 days (n = 3–6). Superoxide detection was made using DHE. **(C)** Expression of p22*^phox^* in cells incubated with 5.5 mM glucose (NG), HG, and HG in presence of 1 nM nifedipine. In the top trace, typical Western blots are shown. β-Actin served as loading control. In the bottom trace, a bar graph summarizes the results of 6 independent experiments. (D) Effect of 5 mM NAC on HG-induced SA-β-gal activity (n = 3). *P<0.05, **P<0.01, ***P<0.001 versus NG. ##P<0.01, ###*P*<0.001 versus HG alone.

We have previously demonstrated that increased ROS plays a critical role in endothelial cell senescence caused by high glucose stimuli [12], [13], [18]. The detection of ROS was performed after staining HUVECs with CM-H_2_DCFDA. CM-H_2_DCFDA is a chloromethyl derivative of H_2_DCFPA, useful as a cell-permeable indicator for the presence of ROS in cells. The cells incubated with high glucose displayed a significant increase in intracellular fluorescence. The addition of H_2_O_2_ led to a further increase in fluorescence of H_2_DCFDA, reflecting CM-H_2_DCFDA having been originally used as an H_2_O_2_ indicator [19]. Nifedipine significantly prevented the increase in ROS generation under high glucose conditions (Figure 3A). The best known ROS are superoxide anion and hydroxyl radical in addition to H_2_O_2_. Superoxide was measured using the oxidative fluorescent dye DHE, which is a widely used sensitive superoxide probe. The high glucose incubation slightly but significantly increased ethidium (i.e., oxidized DHE) fluorescence. This high glucose-induced increase in superoxide was unaffected by nifedipine. The NO donor sodium nitroprusside was used as a reference and it markedly reduced the fluorescence possibly due to stoichiometric interaction between superoxide and NO (Figure 3B). NADPH oxidase is one of the most important sources of superoxide in vascular cells and p22^phox^ is a critical component of the superoxide-generating NADH/NADPH oxidase system [20]. Despite the report showing endothelial-dependent p22^phox^ up-regulation induced by high glucose [21], high glucose did not substantially alter p22^phox^ expression in HUVECs regardless of whether nifedipine wa**s** given (Figure 3C). In similar to nifedipine, NAC, an ROS scavenger, was significantly effective in reducing SA-β-gal activity in a high glucose environment (Figure 3D).

### Nifedipine-induced anti-senescence action in endothelial cells requires the presence of eNOS

**Figure 4:**
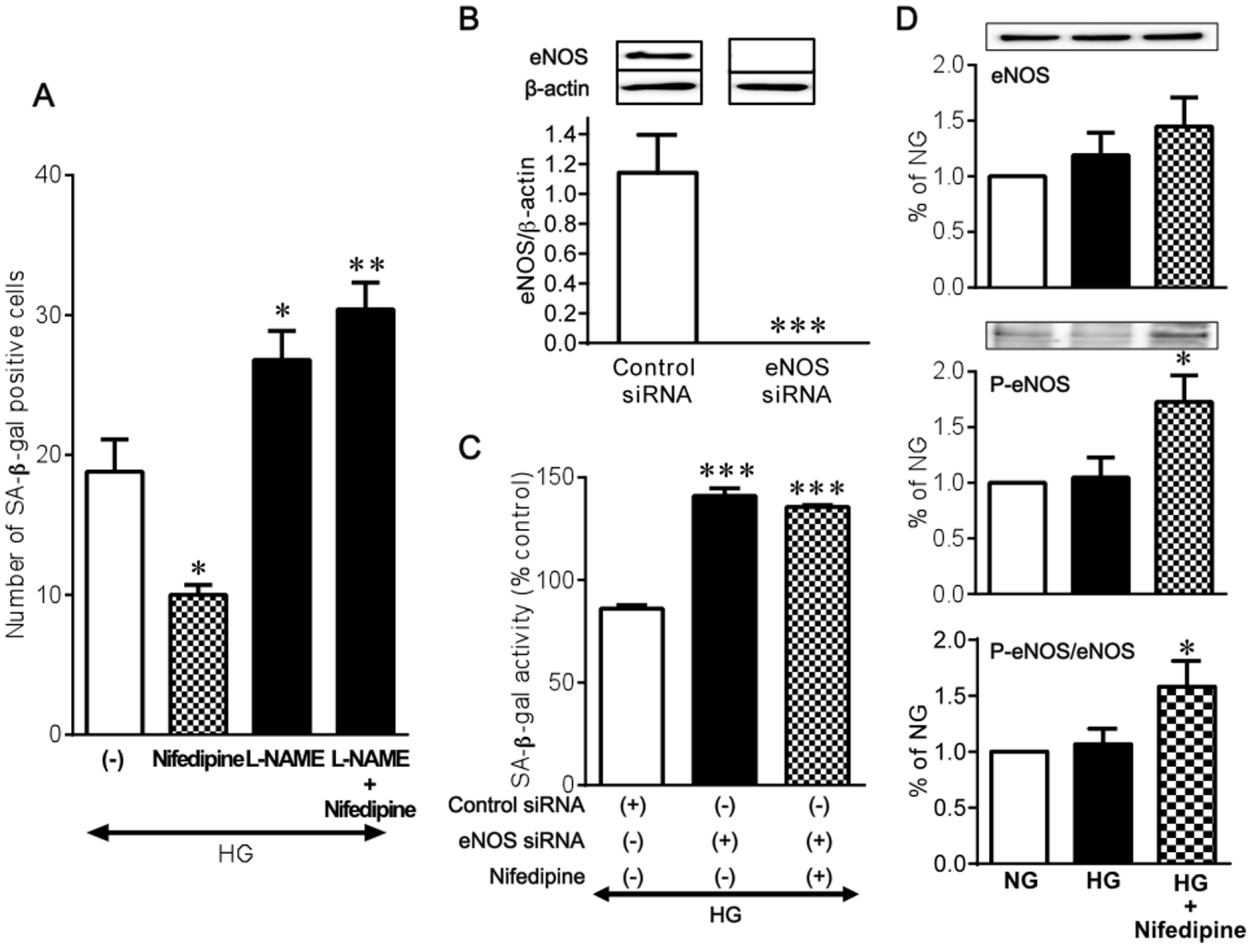
Role of eNOS in the effect of nifedipine on high glucose-induced cellular senescence in HUVECs. **(A)** Influence of 1-NAME on the effect of 1 nM nifedipine on HG (22 mM glucose)-induced increase in SA-β-gal positive cells (n = 5). **(B)** Transfection of eNOS siRNA effectively eliminated eNOS protein expression (n = 3). **(C)** Influence of eNOS siRNA transfection on the effect of 1 nM nifedipine on HG-induced increase in SA-β-gal activity (n = 3). **(D)** Effect of 1 nM nifedipine on eNOS expression and phosphorylation at Ser-1177 under HG conditions (n = 6). *P<0.05, **P<0.01, ***P<0.001 versus HG alone.

We have previously shown that eNOS plays a pivotal role in the regulation of the senescence program in endothelial cells [12], [13], [18]. In the presence of L-NAME, nifedipine failed to reduce SA-β-gal positive cells (Figure 4A). To further to define the involvement of eNOS in the anti-senescence action of nifedipine in endothelial cells, we used siRNA to specifically ablate eNOS mRNA in HUVECs. Our siRNA which was designed to target eNOS successfully silenced endothelial expression of eNOS protein compared with that of the negative control siRNA 72 hours after transfection (Figure 4B). Transfection of eNOS siRNA resulted in a further increase in SA-β-gal activity under high glucose. Again, nifedipine showed no reducing effect on SA-β-gal activity when the ablation of eNOS by siRNA was performed (Figure 4C). Under high glucose conditions, basal eNOS phosphorylation level at Ser-1177 was substantially the same as that obtained under normal glucose. Nifedipine significantly increased eNOS Ser-1177 phosphorylation in a high glucose environment (Figure 4D). Akt is well known to mediate activation of eNOS [22]. Furthermore, eNOS phosphorylation by AMPK has been observed in a variety of conditions, including treatment with metformin [23], [24]. However, treatment with nifedipine showed no apparent up-regulation of the phosphorylation levels of Akt and AMPK in HUVECs exposed to high glucose (Figure S3).

## Discussion

In the present study, we demonstrated that the calcium channel blocker, nifedipine significantly inhibited vascular endothelial cell senescence. Thus, nifedipine prevented the increase in the number of SA-β-gal positive cells and turned the decreased telomerase activity to normal in HUVECs exposed to high glucose. Since such anti-senescence effects were observed with other calcium channel blockers, including amlodipine and benidipine (Hayashi, unpublished observations), these can be considered as a class effect of calcium channel blockers. However, the anti-senescence effect of nifedipine was not related to increasing its concentrations. Moreover, the finding that increasing medium concentration of Ca^2+^ led to a rather significant reduction in SA-β-gal activity under both normal and high glucose conditions suggests that the beneficial effect of nifedipine on endothelial cell senescence cannot be solely attributed to its calcium channel blocking action. Other antihypertensive agents, such as a β_1_-adrenergic blocking agent and an angiotensin-converting enzyme inhibitor, were found to be without effect on endothelial cell senescence.

Recent studies have provided increasing evidence that high glucose accelerates endothelial cell senescence [12]–[14]. In addition, we found that angiotensin II and oxLDL were inducers for endothelial cell senescence. As senescent vascular endothelial cells are linked to atherosclerosis [7], [8], our finding is in line with the idea that angiotensin II and oxLDL are each critical factors in atherogenesis. However, the effects of angiotensin II and oxLDL were less pronounced as compared with high glucose. We do not have a clear understanding of this difference, but may be related to the possibility that the mechanisms whereby angiotensin II and oxLDL accelerate and worsen atherosclerosis are more multifactorial.

Our previous studies have demonstrated that endothelial cellular senescence caused by high glucose stimuli is associated with an increase in ROS and a decrease in eNOS-derived NO [12], [13], [18]. In this study, treatment with nifedipine significantly reduced ROS generation in human endothelial cells exposed to high glucose. Although high glucose resulted in a small increase in superoxide, this superoxide generation was unaffected by nifedipine, suggesting that nifedipine reduced ROS not involving superoxide. In addition, high glucose exposure did not up-regulate endothelial expression of p22*^phox^*, which is a critical component of the superoxide-generating NADH/NADPH oxidase system [21]. On the other hand, we found that nifedipine significantly increased eNOS Ser-1177 phosphorylation in HUVECs under high glucose conditions. Nifedipine failed to activate Akt and AMPK. This suggests that nifedipine can activate basal eNOS activity independently activation of Akt and AMPK. Importantly, inhibition of eNOS with L-NAME completely negated the anti-senescence effect of nifedipine. Furthermore, the ablation of eNOS by siRNA showed no inhibitory effect of nifedipine on endothelial cell senescence. These findings point to the critical need for eNOS in the nifedipine anti-senescence action. Taken together, the beneficial effects of nifedipine on high glucose-induced endothelial cellular senescence may be, at least in part, involve its actions on eNOS and ROS other than superoxide. A causal link between ROS generation and eNOS activity requires further investigation, although our preliminary study showed that high glucose-induced ROS generation in HUVECs was unchanged by the ablation of eNOS by siRNA.

Calcium channel blockers are a widely used group of antihypertensive agents because they lower blood pressure mainly through vasodilation and reduced peripheral resistance. Although hypertension is a driver of the development of atherosclerosis underlying cardiovascular diseases, the benefits of calcium channel blockers, and in particular 1,4-dihydropyridines, for the progression of atherosclerosis have been indicated beyond their blood pressure-lowering effects. Many dihydropyridine calcium channel blockers have been shown to suppress the progression of atherosclerotic lesion of formation in atherosclerotic model animals [25]–[30]. Importantly, most of these studies have revealed that calcium channel blockers at the doses used did not affect blood pressure. The concentration of nifedipine used in our present study (1 nM) is in the range of clinically relevant concentrations found in plasma of patients treated with this drug [31]–[33]. It should be noted, however, that the concentrations above and below 1 nM were found to have a limited effect on endothelial cell senescence.

Calcium channel blockers have demonstrated anti-atherogenic properties in clinical studies, showing slowed progression and decreased formation of new lesions in treated patients [34]–[36]. The development of atherosclerosis may be mediated by endothelial injury, activation of macrophages, and abnormalities of vascular smooth muscle cell function. Possible mechanisms of the anti-atherosclerotic effects of dihydropyridine calcium channel blockers have been proposed to include their ability to protect against endothelial injury, inactivate macrophages, and to improve smooth muscle cell abnormalities [37].

This study represents the first report that the calcium channel blocker nifedipine inhibited endothelial cell senescence in a high glucose environment. Our finding provides new insight into the mechanisms by which calcium channel blockers may be useful in preventing the development of atherosclerosis in diabetes. However, additional work using atherosclerotic model animals may be needed to establish more evidently the significance of the inhibition of vascular endothelial cell senescence by calcium channel blockers in their anti-atherosclerotic actions.

## Supporting Information

Figure S1 Lack of effect of 1 nM nifedipine on SA-b-gal activity(A) and telomerase activity (B) under normal glucose (NG) conditions (n = 3–4).
(TIFF)

Figure S2 **Effect of high Ca^2+^ on SA-b-gal activity in HUVECs under normal glucose (NG) and high glucose (HG) conditions.** Cells were incubated with 5.5 mM or 22 mM glucose for 3 days in the presence of normal (1.8 mM) or high (3.6 mM) Ca^2+^ (n = 3). *P<0.05 versus NG alone. #P<0.05 versus HG alone.
(TIFF)

Figure S3 **Western blots showing no apparent up-regulation of phosphorylation levels of Akt and AMPK in HUVECs under high glucose (HG) conditions in the presence of 1 nM nifedipine.** b-actin served as loading control. This experiment was reported twice.
(TIFF)
