# Supplementary figures and images for: eNOS-Dependent Antisenscence Effect of a Calcium Channel Blocker in Human Endothelial Cells
Source: PLoS One. 2014 Feb 10;9(2):e88391. doi: 10.1371/journal.pone.0088391 (PMC3919771; doi:10.1371/journal.pone.0088391)

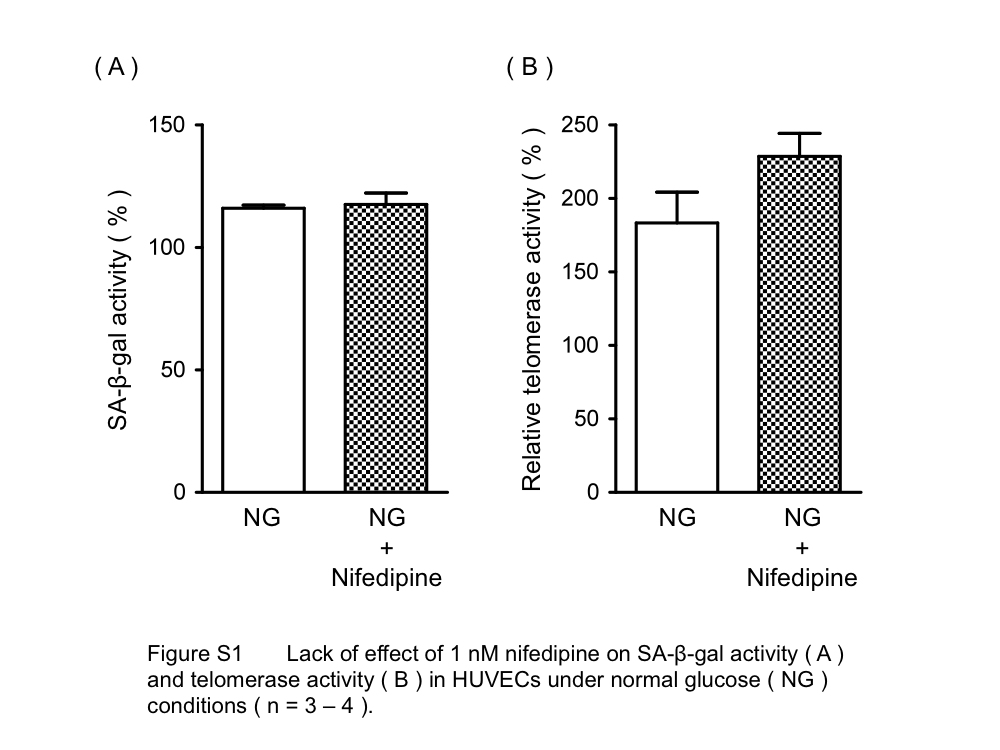

Supplement: Figure S1 — Lack of effect of 1 nM nifedipine on SA-b-gal activity(A) and telomerase activity (B) under normal glucose (NG) conditions (n = 3–4). (TIFF) [file pone.0088391.s001.tiff]

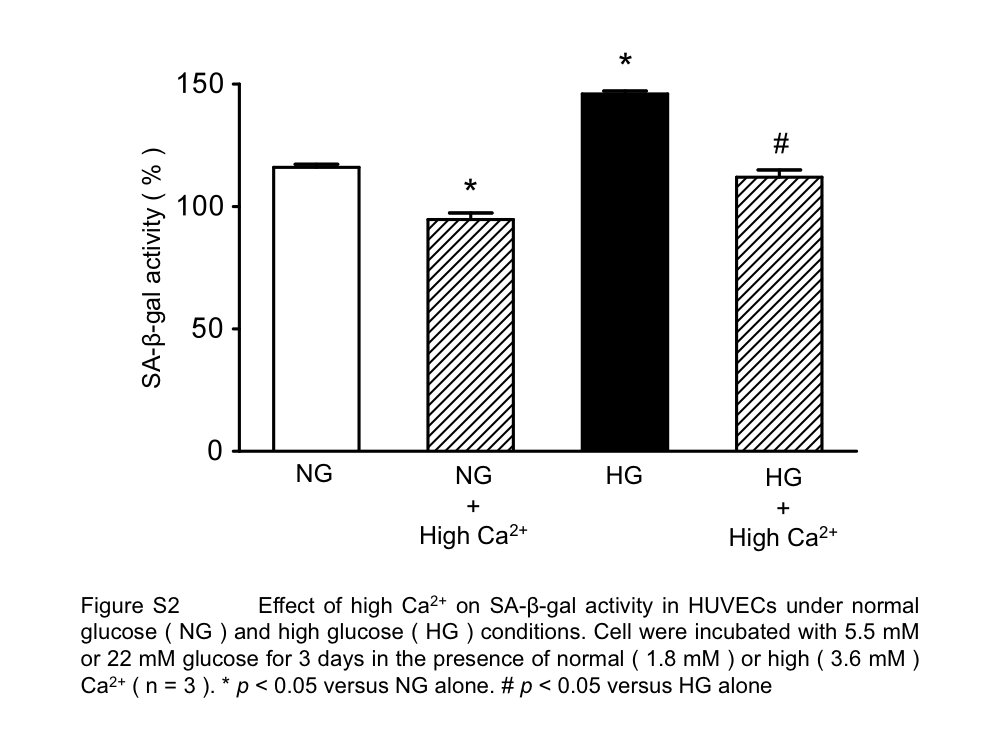

Supplement: Figure S2 — Effect of high Ca2+ on SA-b-gal activity in HUVECs under normal glucose (NG) and high glucose (HG) conditions. Cells were incubated with 5.5 mM or 22 mM glucose for 3 days in the presence of normal (1.8 mM) or high (3.6 mM) Ca2+ (n = 3). *P<0.05 versus NG alone. #P<0.05 versus HG alone. (TIFF) [file pone.0088391.s002.tiff]

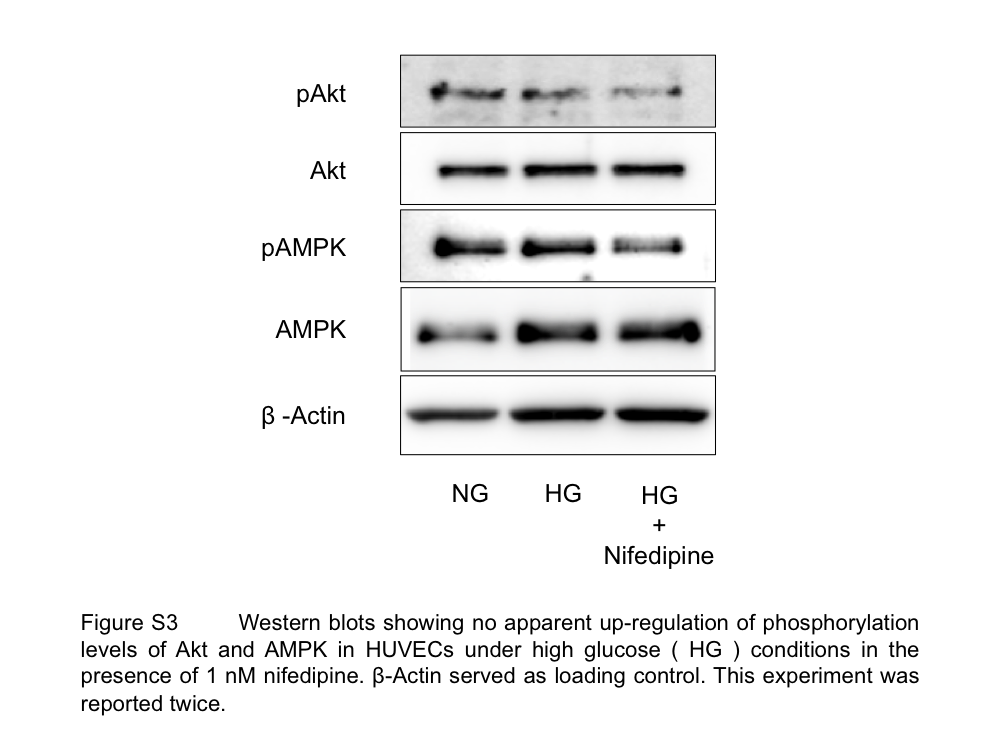

Supplement: Figure S3 — Western blots showing no apparent up-regulation of phosphorylation levels of Akt and AMPK in HUVECs under high glucose (HG) conditions in the presence of 1 nM nifedipine. b-actin served as loading control. This experiment was reported twice. (TIFF) [file pone.0088391.s003.tiff]
